# Using Ethnographic Learning Circle (ELC) to Transform Funds of Knowledge (FoK) into Curricular Assets in Medical Education

**DOI:** 10.5334/pme.2073

**Published:** 2025-12-22

**Authors:** Nurfarahin Nasri, Khairul Azhar Jamaludin, Nurfaradilla Mohamad Nasri

**Affiliations:** 1 Faculty of Education, Universiti Kebangsaan Malaysia, Bangi, Selangor, Malaysia

## Abstract

**Background & Need for Innovation:**

Medical education must prepare students to navigate diverse cultural, linguistic, and community contexts. In Malaysia, students’ funds of knowledge (FoK) are often marginalized in curricula, despite their potential to inform curriculum design. Traditional didactic norms reinforce educators as primary knowledge sources, leaving students’ knowledge and lived experiences underutilized.

**Goal of Innovation:**

The Ethnographic Learning Circle (ELC) was designed to transform and generate FoK-informed curricular assets.

**Steps Taken for Development and Implementation of Innovation:**

ELC engaged 30 Year 3 medical students during community medicine postings. Students first participated in structured community walkthroughs, documenting observations using a four-component FoK template (place, factors, impact, reflections). Subsequently, two 90-minute facilitated circle discussions guided students in collectively reframing their FoK into curricular assets, using an asset–deficit rubric to ensure student-centered reflection.

**Evaluation of Innovation:**

Using a modified Fuzzy Delphi Method, an expert panel reached a 90% consensus that the FoK-informed case study produced through ELC met expectations for transferability, context-specificity, novelty, and curricular alignment. Post-ELC student discussions highlighted enhanced engagement, joy in learning, and more holistic understandings of community health.

**Critical Reflection on your Process:**

Five key lessons included the need for structured student preparation, trained facilitators, sufficient time, assessment alignment, and effective transferability. ELC offers a practical, effective model for embedding FoK-informed curricular assets into medical education.

## Background & Need for Innovation

Medical education aims to equip students with scientific and clinical competence as well as the skills to navigate the complex realities of healthcare. This need is particularly urgent worldwide, where health systems often face the dual burdens of complex disease patterns alongside highly diverse cultural, linguistic, and socioeconomic contexts [1]. These realities underscore the importance of curricula that integrate community knowledge and lived experience into medical learning.

In Malaysia, the 2022 Standards for Undergraduate Medical Education specifically emphasize scientific and professional competence while promoting responsiveness to local contexts [2]. For this reason, teaching sessions often address issues such as insulin titration during Ramadan, herb–drug interactions, or multilingual consent processes. However, these initiatives rely on individual educators’ efforts as they are not systematically embedded in curricula [3]. In many cases, educator-centered teaching approaches can make it difficult to incorporate students’ own knowledge [4]. As a result, students may find fewer opportunities to connect their experiences with formal learning and real-world practice.

The Funds of Knowledge (FoK) concept can address these challenges as it recognizes students’ knowledge as a valuable foundation for learning [5]. In medical education, FoK extends the goals of cultural competence and social accountability by understanding how students’ community knowledge can directly inform curriculum design. However, FoK alone is insufficient; it must be purposefully transformed into curricular assets to prevent it from being treated as incidental anecdotes [6].

Accordingly, the Ethnographic Learning Circle (ELC), representing our core innovation, was developed to address this gap. ELC provides a structured pedagogy to enrich and transform students’ FoK into curricular assets. ELC is grounded in two complementary principles. Critical pedagogy positions students as contributors to teaching and learning [7]. It also encourages students to engage in dialogue and reflection, enabling them to question assumptions and co-construct knowledge drawn from their lived experiences [8]. While ethnographic pedagogy guides the systematic observation, documentation, and interpretation of FoK for curricular use [9]. Ethnographic pedagogy allows students to critically analyze community practices and translate these insights into structured, contextually relevant learning materials [10].

## Goal of Innovation

Existing practices such as reflective essays often emphasize individual self-awareness, whereas cultural competence training focuses on cultural differences [11,12]. Similarly, while community postings offer valuable real-world exposure, they lack mechanisms to transform those experiences into structured learning resources [13].

Unlike prior practices, the ELC first broadened students’ FoK through community walkthroughs and then transformed them into curricular assets through facilitated circle discussions. For medical educators, the ELC provides a practical framework for developing authentic, contextually grounded learning materials that align with existing curriculum structures while enhancing relevance to students and the communities they serve.

## Steps taken for Development and Implementation of Innovation

The ELC was implemented in Malaysia during a Year 3 Community Medicine posting involving 30 medical students. The posting aims to develop students’ understanding of community health, cultural practices, and patient-centered communication. The ELC was intentionally designed to align with the posting’s learning outcomes, namely applying community-based knowledge to clinical reasoning, recognizing culturally relevant health behaviors, and practicing respectful communication in diverse contexts.

### Pre-implementation Phase: Community Walkthroughs

The pre-implementation phase aimed to enrich students’ FoK by engaging them directly with community contexts through community walkthroughs. Students worked in groups of five to observe and document health-related practices in local households and community settings. Table 1 below outlines the principle and steps taken in this phase.

**Table 1:** Practical steps in the pre-implementation phase aligned to ethnographic pedagogy.


PRINCIPLE		

Ethnographic pedagogy • Used in community walkthroughs to enrich and organize FoK systematically • Helped in the design of FoK template to ensure observations are comprehensive and organized		

**STEPS**	**DESCRIPTION**	**RATIONALE**

Step 1: Group formation and consent	Assign and divide students into small groups	Ensures diverse FoK, manageable discussion and equitable participation

	Obtain informed consent from all students	Promotes ethical participation and sets the stage for responsible engagement during the walkthroughs

Step 2: Design of FoK template and briefing - Design takes one or two hours - Template is in printed form	Design the FoK template to include four components: Place: Description of the site Factors: Cultural, religious, or economic influences on practices Impact: Implications for care-seeking or treatment adherence Discussion notes: Reflections linking observations to potential clinical practice	Structures FoK to ensure comprehensive documentation

	Provide a structured briefing covering: Purpose of the walkthroughs Types of practices to observe Dissemination of FoK template Instructions for documenting observations using the FoK template	Ensures students understand the objectives of the walkthroughs, what to observe, and how to record consistently

Step 3: Community walkthroughs and documentation - Conducted across two weeks	Students visit community sites and complete the FoK template *See Appendix 1 for examples of completed FoK templates*	Enriches and documents FoK systematically


### Implementation Phase: Facilitated circle discussions

The implementation phase aimed to help students collectively reflect their FoK and generate FoK-informed curricular assets through facilitated circle discussions. Table 2 below outlines the principles and the steps taken in this phase.

**Table 2:** Practical steps in the implementation phase aligned to critical pedagogy.


PRINCIPLE		

Critical Pedagogy • Applied in facilitator training to prepare facilitators to recognize students’ FoK as assets, manage power dynamics, and facilitate circle discussions effectively • Used during circle discussions to generate FoK-informed curricular assets		

**STEPS**	**DESCRIPTION**	**RATIONALE**

Step 1: Facilitator training - Delivered by experts in critical pedagogy - 8 hours	Two facilitators trained in participatory techniques, balanced dialogue, and asset–deficit framing Included role-play and mock discussions for practice	Trains facilitators with skills to center on students’ FoK

Step 2: Circle discussions - 2 × 90 minutes	All groups present their completed FoK templates Facilitators use open-ended prompts and the asset–deficit rubric to guide reflection and discussion Students generate FoK-informed curricular assets *See Appendix 2 for the application of asset–deficit rubric*	Provides a structured platform for students to critically analyze their FoK and with facilitator support, generate curricular assets.


Through the ELC, different forms of FoK-informed curricular assets were developed such as clinical communication module on negotiating consent in family-mediated contexts and a case study on the culturally sensitive integration of traditional practices.

## Evaluation of Innovation

The evaluation focused on both the process and outcomes of the ELC. For the outcome evaluation, we focused on one key product of the ELC, the FoK-informed case study, as illustrated in Table 3. The case was submitted to an expert panel of curriculum specialists and medical educators, who evaluated it using a modified Fuzzy Delphi Method. The panel assessed the case against four criteria: transferability, context-specificity, novelty, and curricular alignment. Across two rounds, a 90% consensus was achieved that the case met or exceeded expectations across all dimensions. Reviewers emphasized that although the scenario was localized, the communication strategies were transferable to a wide range of multicultural settings. They also noted the case’s authenticity, pointing out that it directly reflected lived realities from the community. The innovation was recognized as lying not in the role-play itself but in generating curricular assets that translated community knowledge into teaching materials aligned with established communication competencies.

**Table 3:** Case study on negotiating consent in family-mediated contexts.


SCENARIO

A 7-year-old Malay child is brought to the rural clinic with persistent fever. The mother reports that before coming to the clinic, the family consulted a *bomoh* (spiritual healer) who performed rituals and prescribed herbal remedies. The grandmother, who holds strong authority in family health decisions, insists that the child continue the herbal treatment alongside any clinic medicines. The father is quiet but defers to the grandmother’s authority. The student-doctor must balance respect for the family’s beliefs with biomedical recommendations, while negotiating consent for paracetamol and follow-up blood tests.

**TEACHING AND LEARNING ACTIVITY**

Structured Role-Play (student-doctor vs. family member actors, rotating roles). Students are instructed to practice communication strategies: 1. Acknowledging the legitimacy of traditional practices without dismissing them (e.g., “I understand many families find comfort in visiting a *bomoh* (spiritual healer)”). 2. Negotiating biomedical and traditional practices by highlighting complementarity rather than conflict (e.g., “These medicines will help with the fever while your herbal remedies continue to provide strength”). 3. Managing family-mediated communication by addressing elders respectfully, inviting the grandmother’s input, and ensuring that consent is negotiated collectively. 4. Using indirect but clear phrasing to avoid confrontation, reflecting local communication norms. After role-play, students write a short reflection linking their communication strategies to what they learned about family and cultural practices during the walkthroughs.

**CURRICULAR VALUE**

Students learn to move beyond generic cultural competence and instead apply FoK to real clinical communication challenges, practicing how to balance biomedical reasoning with cultural sensitivity and family dynamics.


For process evaluation, post-ELC student review discussions indicated that participation in the ELC fostered greater engagement among students. They described the ELC as enjoyable and low-pressure, which encouraged meaningful exchange of their FoK. One student reflected, “It felt different from other classes because we were learning from real experiences, not just from textbooks.” Students also noted that the ELC facilitated more holistic understanding of community health and its relevance to clinical practice.

Overall, following this positive evaluation, the ELC was forwarded to the medical education curriculum committee and is currently under review for formal inclusion in the upcoming semester.

## Critical Reflection

Implementing the ELC revealed challenges across five key areas, namely structured student preparation, skilled facilitation, sufficient time and continuity, assessment alignment and effective transferability. We offer several practical strategies in each area to guide future applications.

### 1. Structured student preparation

Students require explicit preparation before community walkthroughs. Unguided observations often became too descriptive (e.g., “houses are small,” “people sit together at shops”) or overlapped across groups thus limiting the diversity of insights. A focused briefing session should therefore:

- Define objectives (to identify cultural practices and everyday health-related behaviors, not just describe physical settings)
- Clarify what to observe (e.g., traditional healing practices, household health routines, intergenerational caregiving roles)
- Standardize documentation methods through the FoK template to ensure comparability across groups. This scaffolding increases the analytical quality of student observations and makes them a stronger foundation for circle discussions

### 2. Skilled facilitation

Facilitators play a central role in preventing discussions from reverting to one-way teaching. In early sessions, students tended to defer to expert authority, waiting for facilitators to interpret observations. Training must therefore equip facilitators with skills in:

- Using open-ended prompts (“What does this practice tell us about family health priorities?”)
- Encouraging peer-to-peer dialogue rather than lecturer-to-student exchange
- Applying the asset–deficit rubric to redirect deficit statements (“They delay care by going to the *bomoh* (spiritual healer) first”) into asset-oriented reframing (“They seek a trusted healer first; how can that trust be integrated into clinic-based care?”)

In our case, using two facilitators proved valuable as one actively guided reflection, while the other monitored group dynamics, documented insights, and ensured equitable participation. This division of labor prevented dominant voices from taking over and kept discussions on track.

### 3. Sufficient time and continuity

Depth of engagement requires time beyond a single sitting. Two 90-minute sessions generated enthusiasm but often ended just as discussions reached their most productive stage of reframing FoK into curricular assets. Students themselves expressed frustration at the abrupt ending, reporting that “the most important part which is what to do with the insights often felt rushed”. For replication, we recommend embedding micro-ELCs (shorter, recurring sessions across multiple postings) rather than relying on one-off blocks. This allows students to revisit, refine, and deepen their use of FoK without overloading existing curricula.

### 4. Assessment alignment

Students appreciated the novelty of the ELC but questioned its educational weight when outcomes were not formally assessed. Without linkage to assignments or examinations, there is a risk that FoK-informed learning will be perceived as nice but not essential. To enhance legitimacy and sustainability, FoK insights should be tied to assessment formats already in use:

- Incorporate FoK analysis into reflective essays
- Use FoK-derived scenarios in PBL tutorials
- Test application in OSCE stations where communication with families or traditional healers is required

This alignment not only validates FoK-informed curricular assets but signals to students and faculty that it is central, not peripheral to teaching and learning in medical education.

### 5. Effective transferability

While originally developed in Malaysia, the ELC’s underlying pedagogy is adaptable across diverse educational contexts. Successful transfer requires attention to both cultural relevance and resource constraints. Three practical strategies can support transferability:

- Contextualize observation tools to reflect local health beliefs, community dynamics, and communication practices relevant to each setting
- Simplify facilitation formats by using small-group or peer-led discussions, making ELC feasible in resource-limited or rural training environments
- Leverage digital storytelling or online reflection platforms to gather community narratives in multicultural or geographically dispersed classrooms

These adaptations preserve the core pedagogical intent of the ELC while allowing flexible implementation across varied institutional and cultural contexts.

## Conclusion

In conclusion, the ELC is replicable across diverse contexts when structured student preparation, skilled facilitation, adequate time, assessment alignment, and effective transferability are intentionally built into its design. Beyond medical education, it has clear applicability to nursing, pharmacy, and public health programs, particularly in culturally diverse or resource-constrained settings. By embedding FoK as curricular assets, the ELC offers a scalable model for advancing equity and contextual relevance in health professions education.

## Figures and Tables

**Appendix 1:**
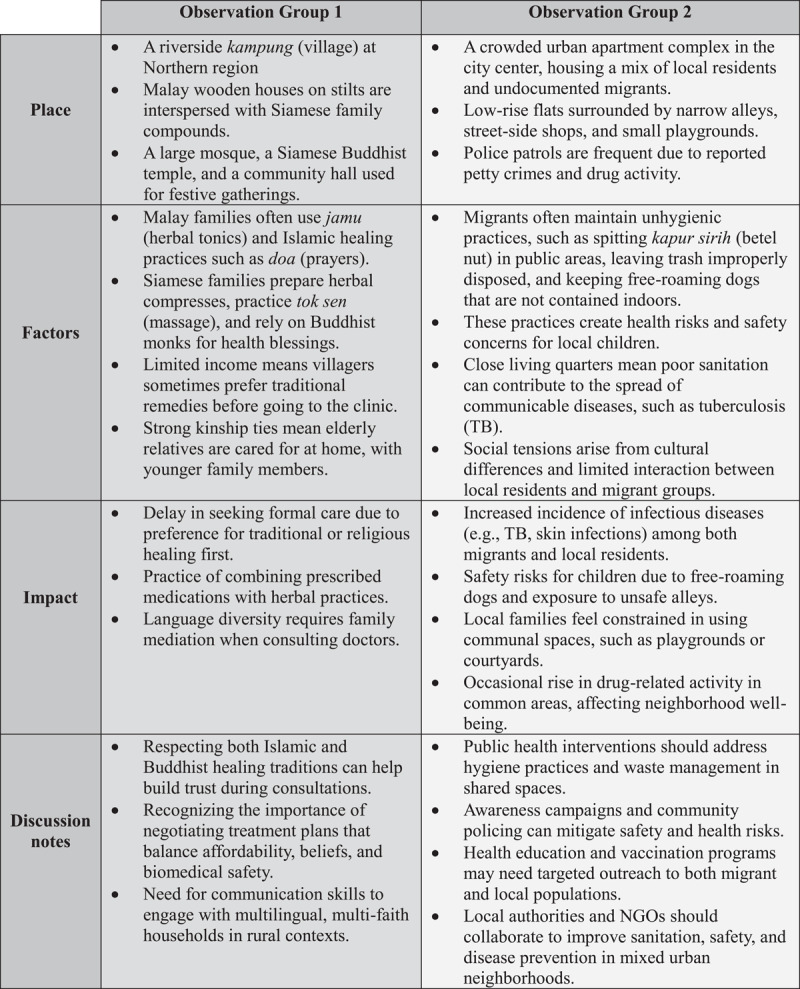
Two observations in the FoK template.

**Appendix 2:**
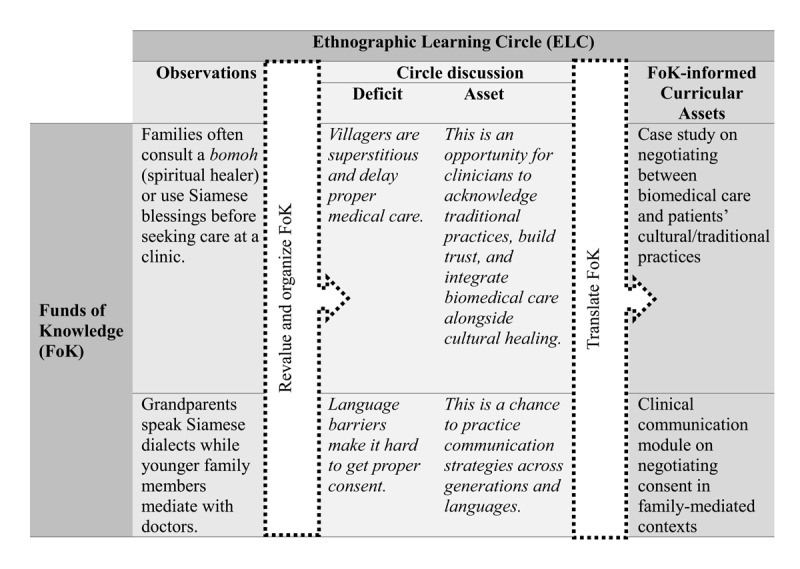
Summary on how asset-deficit rubric can transform FoK into curricular assets.
